# The C2 isthmus screw provided sufficient biomechanical stability in the setting of atlantoaxial dislocation-a finite element study

**DOI:** 10.1186/s12891-024-07470-6

**Published:** 2024-05-29

**Authors:** Minming Lu, Zhenqiang Wang, Bo Yuan, Yifan Tang, Changjiang Gu, Shengyuan Zhou, Xiongsheng Chen

**Affiliations:** Spine Center, Department of Orthopaedics, Changzheng Hospital Naval Medical University, (Second Military Medical University), Shanghai, 200003 P.R. China

**Keywords:** Biomechanics, Isthmus screw, Finite element model, Posterior fixation, Atlantoaxial dislocations

## Abstract

**Background:**

The emerging of the C2 isthmus screw fixation technique is gaining popularity in the setting of atlantoaxial dislocation or other conditions requiring fixation of C2. However, the biomechanical stability of this fixation is poorly understood.

**Purpose:**

To compare and elucidate the biomechanical stability of C2 pedicle screw (C2PS), C2 isthmus screw (C2IS) and C2 short isthmus screw (C2SIS) fixation techniques in atlantoaxial dislocation (AAD).

**Method:**

A three-dimensional finite element model (FEM) from occiput to C3 was established and validated from a healthy male volunteer. Three FEMs, C1 pedicle screw (PS)-C2PS, C1PS-C2IS, C1PS-C2SIS were also constructed. The range of motion (ROM) and the maximum von Mises stress under flexion, extension, lateral bending and axial rotation loading were analyzed and compared. The pullout strength of the three fixations for C2 was also evaluated.

**Result:**

C1PS-C2IS model showed the greatest decrease in ROM with flexion, extension, lateral bending and axial rotation. C1PS-C2PS model showed the least ROM reduction under all loading conditions than both C2IS and C2SIS. The C1PS-C2PS model had the largest von Mises stress on the screw under all directions followed by C1PS-C2SIS, and lastly the C1PS-C2IS. Under axial rotation and lateral bending loading, the three models showed the maximum and minimum von Mises stress on the screw respectively. The stress of the three models was mainly located in the connection of the screw and rod. Overall, the maximum screw pullout strength for C2PS, C2IS and C2SIS were 729.41N, 816.62N, 640.54N respectively.

**Conclusion:**

In patients with atlantoaxial dislocations, the C2IS fixation provided comparable stability, with no significant stress concentration. Furthermore, the C2IS had sufficient pullout strength when compared with C2PS and C2SIS. C2 isthmus screw fixation may be a biomechanically favourable option in cases with AAD. However, future clinical trials are necessary for the evaluation of the clinical outcomes of this technique.

**Supplementary Information:**

The online version contains supplementary material available at 10.1186/s12891-024-07470-6.

Atlantoaxial dislocation (AAD) can resulting in joint dysfunction, nerve compression or even devastating consequence due to trauma, degeneration, congenital deformity, inflammation and other factors. The aim of surgical treatment is to relieve the nerve compression thus to prevent progressive neurological dysfunction and to restore the stability of the atlantoaxial joint [1]. Currently, the mainstream surgical approach is the posterior atlantoaxial screw and rod fixation technique (i.e., Harms' technique) which was modified by Harms et al. [2] on the basis of Goel et al. [3] in 2001. Since the C2 pedicle screw penetrates through the posterior, middle and anterior column of the spine, it shows excellent biomechanical stablity and becomes the most preferred choice for posterior fixation in the setting of atlantoaxial dislocation [4, 5]. However, C2 pedicle screw is not applicable in cases of developmental defects of the pedicle, small pedicle or high riding vertebral arteries (HRVA) [6, 7]. Also, the incidence of vertebral artery injury with pedicle screw is as high as 8.5% [8]. In the meantime, there are some other disadvantages to the insertion of pedicle screw as well. For one thing, as the entry point of the C2 pedicle screw was the cranial and medial quadrant of the isthmus surface of C2, with the trajectory 20 to 30 cranially and medially, surgeons have to perform a wider dissection of the paraspinal muscles eventually leading to soft tissue retraction to achieving a certain mediolateral angle. For another, in facing of vertical and angulated dislocation, the short anteroposterior and vertical distance between the heads of the C1 and C2 screw can result in a lack of space for reduction [9].

In 2005, Bristol et al. [10] firstly presented a case of Effendi II cervical fracture treated with isthmus/pars interarticularis screw, which not only resulted in satisfactory reduction of the fracture but also sacrificed no spinal motion, with both excellent fracture healing and solid fixation at the 6-month postoperative follow-up. Since then, an increasing number of scholars have been applying this technique in a variety of diseases requiring fixation of C2 in the craniocervical junction area, and achieved satisfactory outcomes [11–13]. However, to the best of our knowledge, there have been no biomechanical studies of this approach. In view of this, the author conducted a finite element study to investigate the biomechanical performance differences in terms of stability, internal fixation stress and pullout strength among the three types of fixation, namely C1PS-C2PS, C1PS-C2IS and C1PS-C2SIS, to provide more details for clinical decision making.

## Materials and methods

This study have been performed in accordance with the ethical standards in the 1964 Declaration of Helsinki.This study was approved by our institutional review board (approval number: 2017SL015). The requirement for informed consent from the participants was waived because of its retrospective nature. For the construction of the finite element model, a healthy male volunteer (38 years old, height 173 cm, weight 74 kg), with no history of cervical spine trauma or surgery, imaging examination ruled out cervical spine fracture, inflammation, tumour, deformity and degenerative disease was included. A 64-row spiral CT scanner (GE, USA) was used at the Imaging Medicine Centre to scan the upper cervical spine from C0-C3, with scanning conditions set at 140 kV and 200 mA and a slice thickness of 0.625 mm. Finally, we obtained 214 tomographic images. The DICOM data was imported into Mimics21.0 (Materialise Company, Belgium) and a three-dimensional model was generated. Subsequently, the spine model was imported to Geomagic Studio 2014 (Raindrop Company, America) and to generate a finite element model (FEM) for analysis. To mesh the model and construct the main ligaments, HyperMesh 2019 (Altair Engineering, Inc., Troy, Michigan, USA) was utilized. Finally, MSC.Patran2019 (NASA Company, America) was used for model assembly, material property definition and finite element analysis. The intact model is consisted of the occiput, cervical vertebraes, intervertebral disc, facet joints and ligaments attached to the craniocervical junction area (Table 1). The thicknesses of the cortical bone and cartilage endplate were 1 mm and 0.5 mm respectively. The intervertebral disc was composed of the nucleus pulposus, annulus fibrosus and annulus ground substance. The analysis of the parameters was in accordance with the suggestions of a previous study [14, 15]. Bone, disc and cartilage structures were assigned linear elasticity. The assignment of the properties for all elements was as follows (Table 2).

**Table 1:** Ligaments and material properties of the FEMs

Ligament	Location	Stretch:Force(mm:N)	Range(mm:N)
Alar ligament	C0-C2	14:350	(7–21):(130–580)
Apical ligament	C0-C2	10:200	(0–29):(100–300)
Transverse ligament	C0-C2	25:400	(10–40):(360–500)
Anterior atlanto-occipital membrane	C0-C1	19:230	(16–22):(200–250)
Anterior longitudinal ligament	C1-C2	12:280	(5–18):(150–420)
Anterior longitudinal ligament	C2-C3	9:210	(5–13):(100–300)
Joint capsules	C0-C2	12:80	(10–14):(30–120)
Posterior longitudinal ligament	C2-C3	10:80	(0–20):(0–160)
Posterior atlantooccipital membrane	C0-C1	18:80	(15–21):(60–100)
Ligamentum flavum	C1-C2	9:80	(4–14):(30–200)
Ligamentum flavum	C2-C3	6:90	(5–7):(20–150)
Ligamenta capsulare	C0-C1	10:320	(2–18):(190–450)
Ligamenta capsulare	C1-C2	9:310	(5–14):(170–460)
Ligamenta capsulare	C2-C3	9:210	(4–14):(80–340)
Interspinal ligaments	C0-C3	7:37	(5–9):(35–39)

**Table 2:** Material parameters of the cervical spine

	Young’s Modulus (MPa)	Poisson Ratio (v)	Element Type
Screw or rod	110,000	0.3	TetMesh Tet4
Cortical bone	12,000	0.29	TetMesh Tet4
Cancellous bone	450	0.29	TetMesh Tet4
Cartilage	10	0.3	TetMesh Tet4
End plate	500	0.4	TetMesh Tet4
Back-end structure	3500	0.3	TetMesh Tet4
Fibre ring	4.2	0.45	TetMesh Tet4
Annulus fibers	30(axial direction) 6(lateral direction)	0.016	1D Spring
Nucleus pulposus	1	0.499	TetMesh Tet4
All ligament			1D Spring

### Establishment of unstable and three fixed FEMs

The unstable model due to the removal of transverse ligament was constructed based on the intact model. Then, we created three additional unstable FEMs to simulate the three different atlantoaxial posterior screw fixation techniques. Finally, five models were analyzed and compared: (i) intact, (ii) unstable, (iii) C1PS-C2PS, (iv) C1PS-C2IS and (v) C1PS-C2SIS. To construct the fixed model, the parameters of the screw, rod and nut were referring from Shao et al. [16]. The curvature of the corresponding rods were constructed according to the anatomical morphology of the patient's upper cervical spine. To simplify the model, post-fixation bone grafts were not modelled.

### Surgical procedures

**Fig.1:**
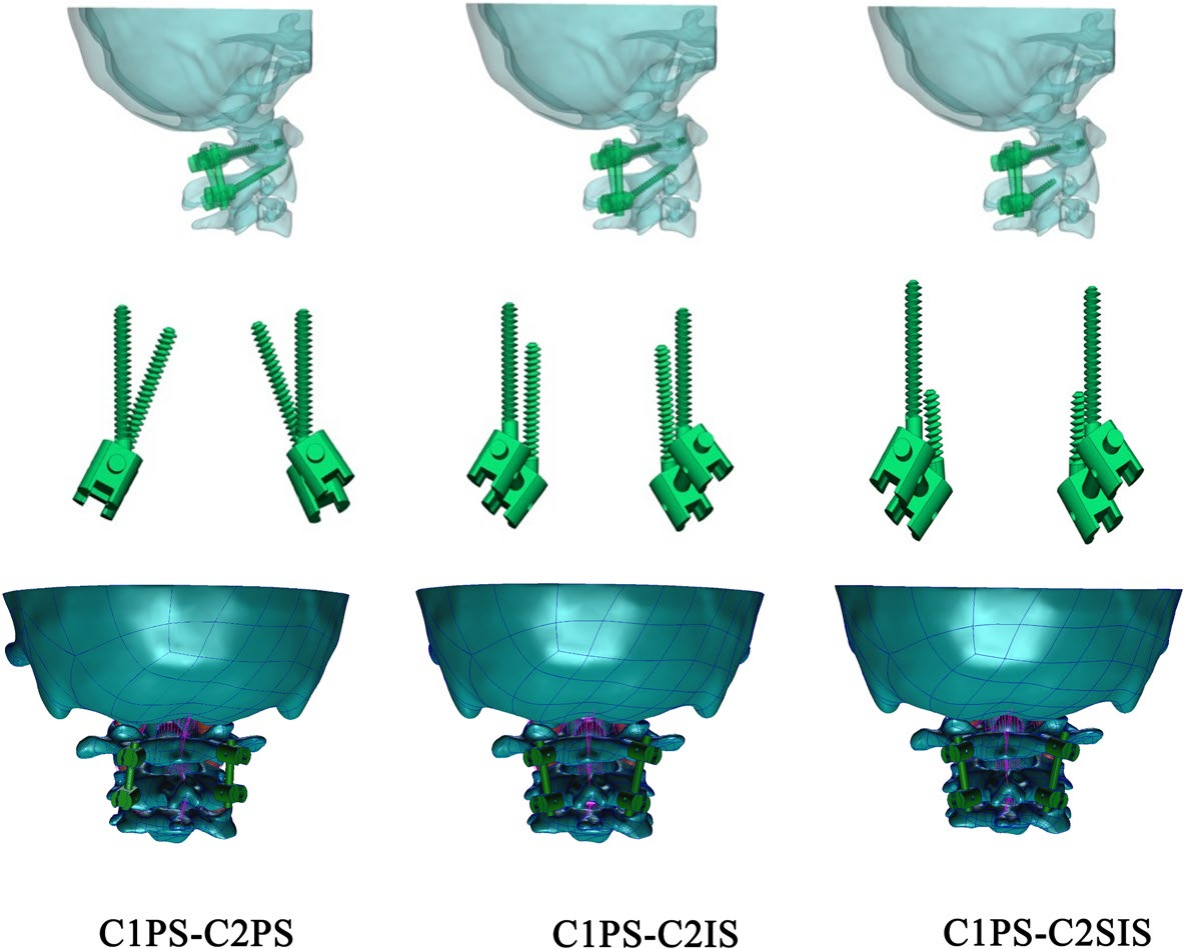
FEMs with three different fixation methods; FEM represent the finite element model

The entry point and trajectory of C2PS was inserted as described in the literiture [4]. The entry point for C2IS was 2–3 mm lateral to the junction of the midline of the C2 lamina and lateral mass, which is more inferior and inward compared with the pedicle screw. The trajectory is placed along the isthmus, and the screw is placed as long as possible into C2 without exceeding the upper articular surface of the C2 at the exiting point. The C2SIS has the same entry point and trajectory with C2IS, which just stopped in the posterior wall of the vertebral artery foramen. In our study, all the screws of C2 were fixed with unicortical. The length of the three types of screw (C2PS, C2IS and C2SIS) was 26 mm, 24 mm and 16 mm respectively with 3.5 mm in diameter (Fig. 1).

### Boundary and loading conditions

The lower surface of the C3 vertebra was constrained in all directions. A pure load of 50N and a torque of 1.5Nm were applied to C0 to simulate the loading conditions of the cervical spine according to studies that had been established in other C0-C2 FE models [17, 18]. To reach the target moment, 10 load steps were applied. The ROM calculated at the end point of the loading cycle was compared with that reported in a human study [19, 20]. Frictional contact interaction for the facet joints was defined as using a coefficient of friction of 0.5 [21]. The interaction between bone and screw was defined as using a coefficient of friction of 0.3 [22]. To only investigate the impact of the fixation methods, we assumed all models shared the same boundary and loading conditions.

### Pullout strength

The load is applied along the longitudinal axis of the screw at the end of the screw. A sudden increase in the displacement of the screw head, i.e. a sudden increase in the slope of the curve, indicates that the screw is loosening and the corresponding loading value is the maximum pullout strength.

## Results

### Validation of the intact FEM

The ROM of the intact models were 11.9°, 10.2°, 4.2° and 27.2° under flexion, extension, lateral bending and axial rotation loading condition respectively, which were 3.9°, 2.9°, 1.8° and 5.3° lower than the unstable model. Our results are in accordance with those of previous both cadaveric and FEA simulation studies [19, 20, 23–25] (Table 3).

**Table 3:** **c**omparision of the ROM with previously published data

Study	ROM (C1-C2)	Flexion	Extension	Lateral Bending	Axial Rotation
Panjabi *et al*^*25*^	Intact model	12.3 ± 2.0	12.1 ± 6.5	3.3 ± 2.3	28.4 ± 4.8
Brolin *et al*^*21*^	Intact model	11.3	14	6.0	23.3
	Intact model	11.9	10.2	4.2	27.8
	Unstable model	15.8	13.1	6.0	33.1
Current study	C2 PS	2.1	1.7	0.55	0.85
	C2 IS	1.8	1.5	0.45	0.75
	C2 SIS	1.9	1.6	0.45	0.8

### ROM of the three fixed FEMs

**Fig.2:**
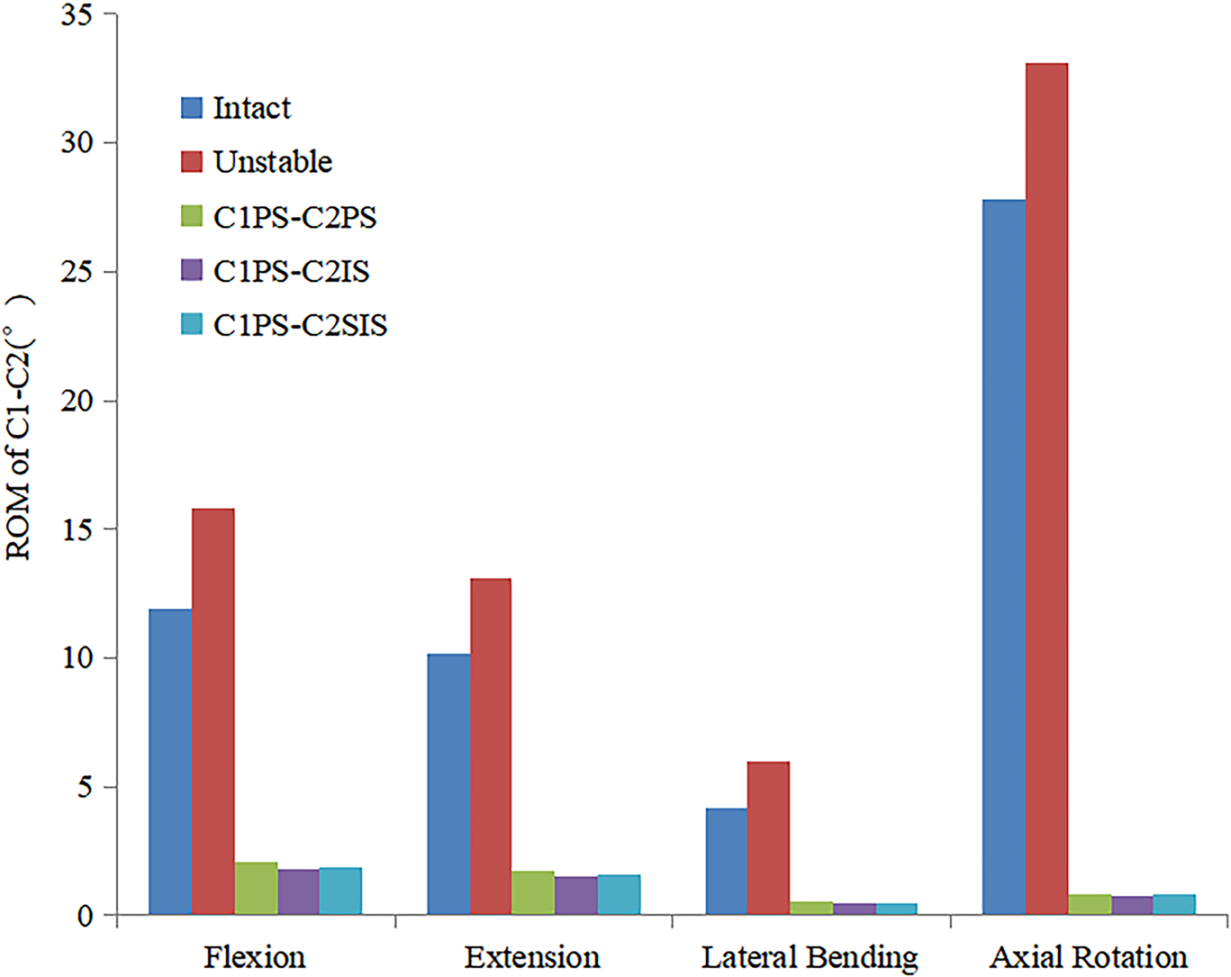
Comparison of the ROM at C1-C2 segment for the FEMs at different state

**Fig.3:**
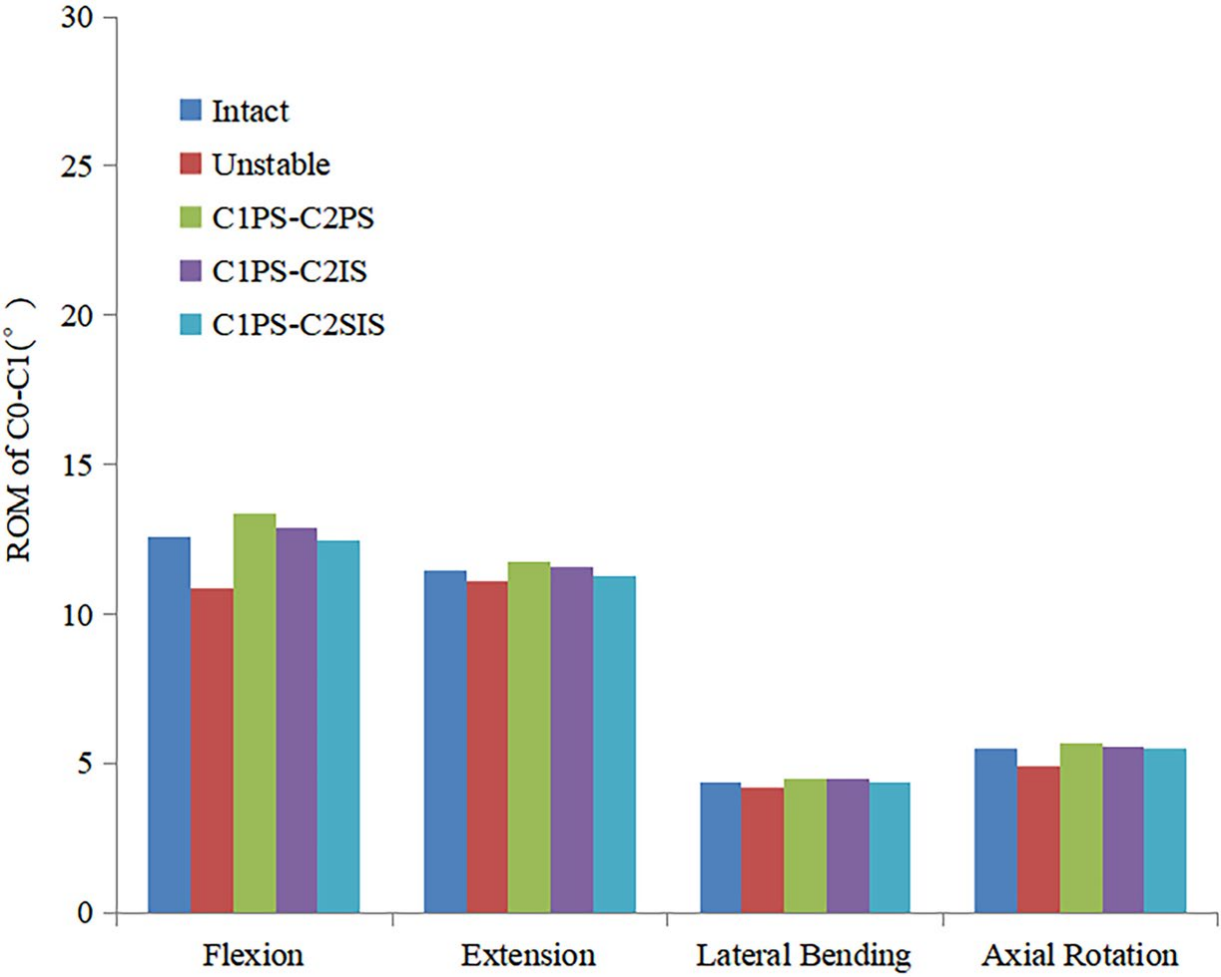
Comparison of the ROM at C0-C1 segment for the FEMs at different state

**Fig.4:**
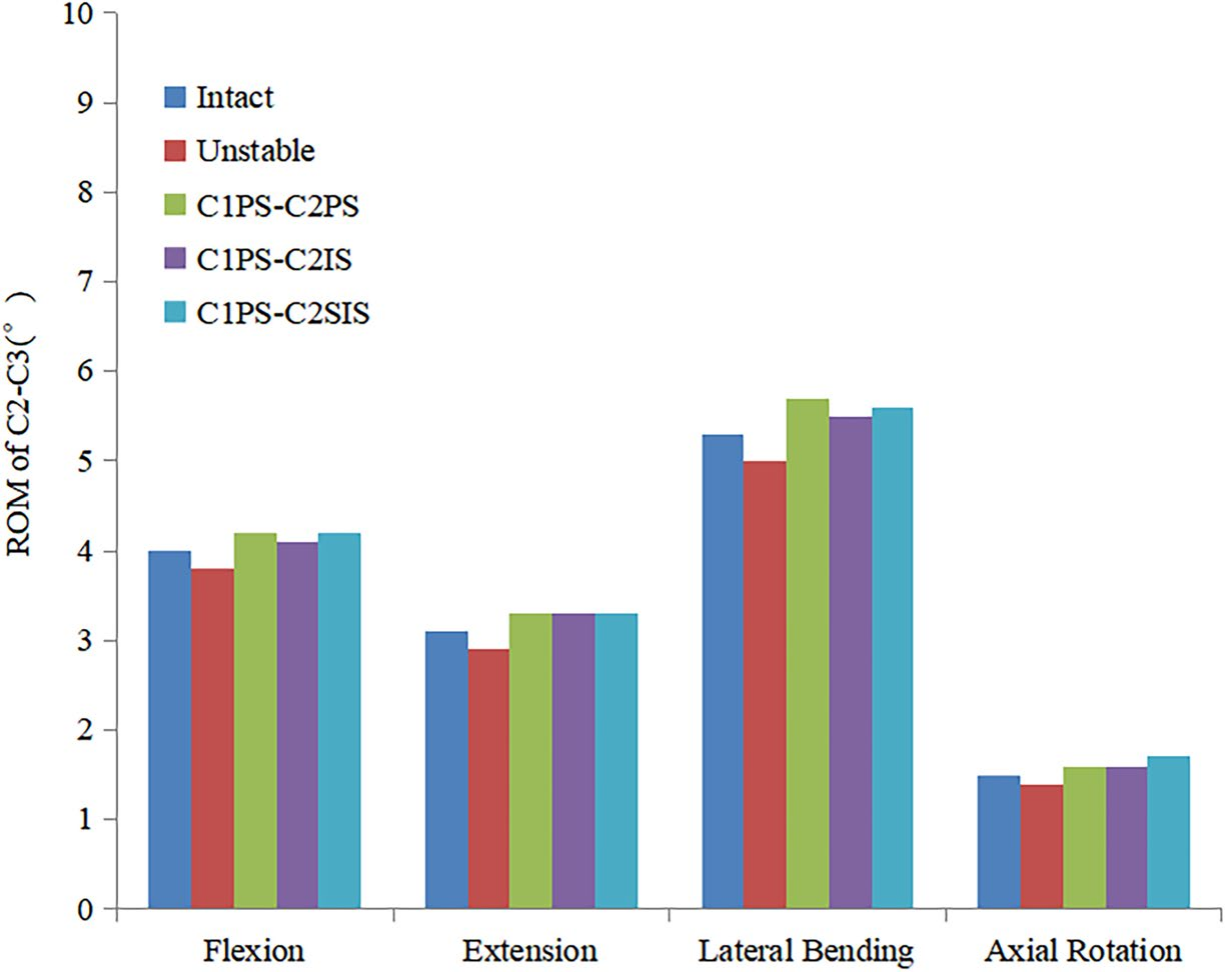
Comparison of the ROM at C2-C3 segment for the FEMs at different state

The ROM at C1-C2 for the C1PS-C2PS, C1PS-C2IS and C1PS-C2SIS models under flexion, extension, lateral bending and axial rotation loading conditions were 2.1°, 1.7°, 0.55°, 0.85° and 1.8°, 1.5°, 0.45°, 0.75°, as well as 1.9°, 1.6°, 0.45°, 0.8° respectively. The ROM at C1-C2 for the C1PS-C2PS, C1PS-C2IS and C1PS-C2SIS models in flexion, extension, lateral bending and axial rotation conditions were reduced by 86.7%, 87.0%, 90.8%, 97.4% and 88.6%, 88.5%, 92.5%, 97.7%, as well as 88.0%, 87.8%, 92.5%, 97.6% respectively (Fig. 2). Additionally, the ROM at C0-C1, C2-C3 segment did not show significant changes at different state (Figs. 3 and 4).

### Stress of the three fixed FEMs

**Fig.5:**
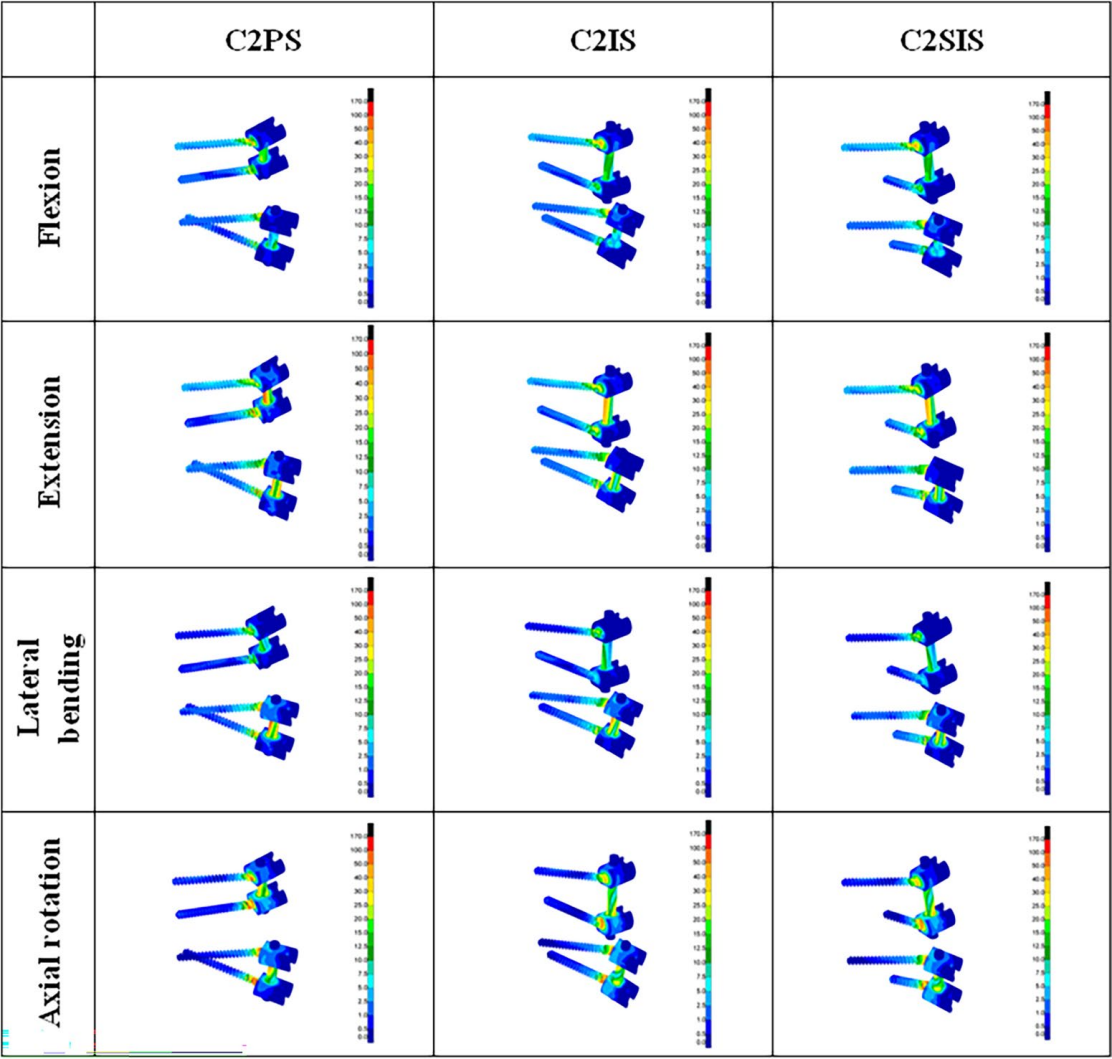
Von Mises stress of the screw-rod constructs

The von Mises stress of the three fixed FEMs, C1PS-C2PS, C1PS-C2IS and C1PS-C2SIS, were different on the screw under four loading conditions. Under flexion, extension, lateral bending and axial rotation, the maximum von Mises stress on the screw for the C1PS-C2PS model were 101.16 MPa, 110.59 MPa, 105.09 MPa and 170.05 MPa respectively. The maximum von Mises stresses on the screws for the C1PS-C2MIS model were 87.98 MPa, 87.56 MPa, 92.1 MPa and 99.16 MPa respectively. The maximum von Mises stresses on the screws for the C1PS-C2SIS model were 88.37 MPa, 88.74 MPa, 92.37 MPa and 119.70 MPa respectively. The maximum von Mises stresses under axial rotation loading conditions were highest on the screw for the three fixations. The stresses of the three models were mainly located in the area of the connection between the screws and the rods (Fig. 5).

### Pullout strength

**Fig.6:**
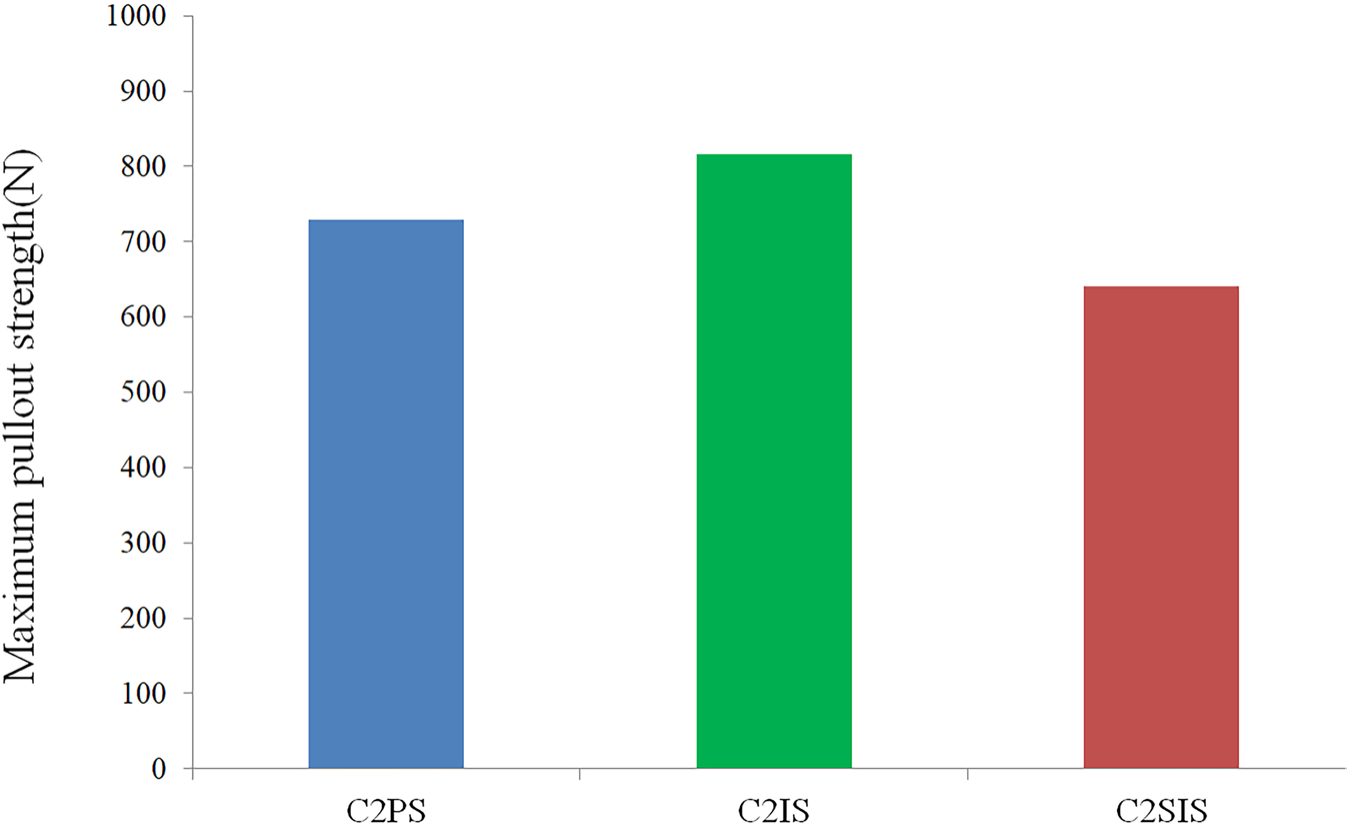
The maximum pullout strength of the three kinds of screw

**Fig.7:**
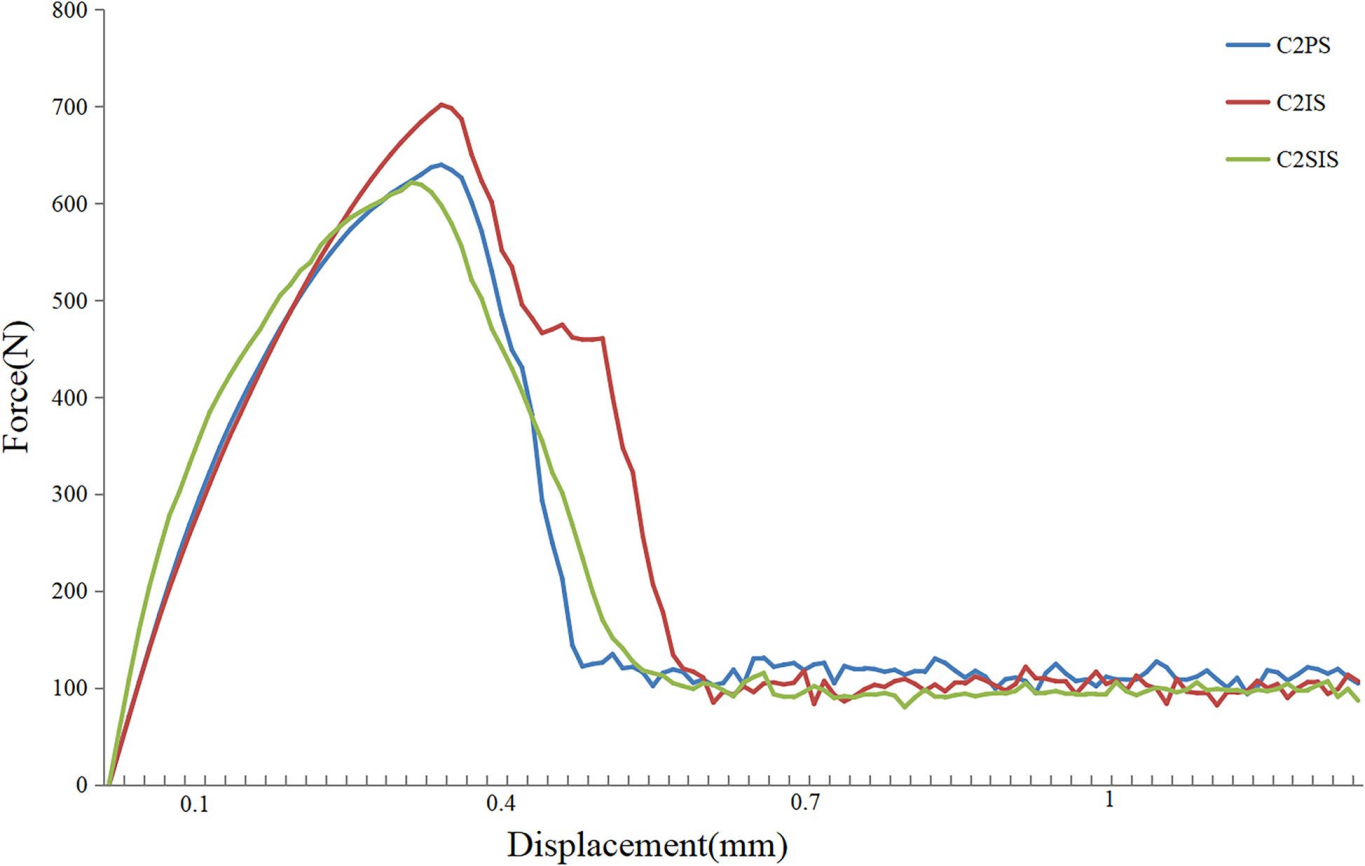
Pullout strength-displacement curve of the three kinds of screw

The maximum screw pullout strength for the three FEMs and screw pullout strength-displacement loading curve are shown in Figs. 6 and 7 respectively.

## Discussion

### Characteristics of the isthmus screw

The isthmus is a small, thin segment of bone that connects the facet joints at the back of the spine [26]. In 2005, Bristol et al. introduced the isthmus screw for the treatment of atlantoaxial instability caused by Effendi II, and achieved satisfactory clinical results [10]. The entry point of isthmus screw was located 2–3 mm lateral to the junction of the midline of the C2 lamina and the lateral mass. The screw was inserted along the direction of the isthmus as long as possible without exceeding the superior articular surface of C2 to achieve maximum screw length. Our initial study found that with the change of mediolateral angle and cephalad angle of the isthmus screw, the length of the screw that could be implanted varied remarkablely. It is relatively safe to place the isthmus screw with a diameter of 3.5 mm and a length at least of 23 mm if the screw is placed at a 10–15° mediolateral angle and 25–35° cephalad angle through the isthmus on both left and right side.

Finite element analysis (FEA) uses mathematical approximations to simulate real spinal structures, and since Belytschko et al. [27] first applied it to the field of spine surgery in 1974, the technique is widely used to analyze the biomechanical properties of a variety of implants in spine [28, 29]. Traditional in vivo mechanical experiments has such problems as difficulties in obtaining specimens, invasive and can not further analyze stress distribution of the implants. FEA has the advantages of being reproducible, time saving and low cost. Therefore, the author applied the finite element method in seeking the biomechanical performance of the C2 isthmus screw.

The results of the study showed that the C1PS-C2IS model indicated the most significant reduction in ROM at all directions under the loading conditions, followed by the C1PS-C2SIS and finally by the C1PS-C2PS. The result seems counterintuitive at first glance, but a closer look reveals that there is something to it. We speculate that it may be related to the following reasons: First, the length of the C2 isthmus screw is 24 mm, which is not significantly shorter than the pedicle screw (26 mm) in this study. Secondly, the greater cephalad angle of the isthmus screw results in a closer proximity to the cortical bone on the upper surface of the C2 vertebral body, similar to the cortical bone trajectory screw which can provide enhanced screw purchase and interface strength independent of trabecular [30]. As to the C2SIS model, it showed higher reduction in ROM compared with C2PS. One of the possible reason for this is that the relatively longer length of the isthmus screws in the present study, and the proximity of the model's screw head to the medial margin of the cortex, similar to the bicortical screw fixation mentioned in a previous study which provides sufficient biomechanical stability compared with C2 pedicle screw [16]. These heterogeneous results suggest that a homogeneous experimental design and condition is necessary, and further clinical studies are needed to validate the results of this study.

In our study, the maximum von Mises stress on the screw for the three internal fixations (C2PS, C2IS, C2SIS) were highest in axial rotation, and lowest in lateral bending. The von Mises stresses are mainly concentrated at the connection of screw and rod, and the results of this study are in consistent with previous studies [16, 31].

As to the pullout strength, the results of our study found that all the three C2 unicortical screws showed comparable biomechanical stability. For one thing, the pullout strength were higher than that in vitro cadaveric studies measured by Su et al. [32] and Dmitriev et al. [33], and the reasons for this may be related to the older age of the patients in the cadaveric specimen experiments, the repeated retrieval and placement of the screws. For another, the maximum pullout strength of the C2 isthmus screw was higher than that of the C2 pedicle screw, which may be related to the fact that the C2 isthmus screw hold the cortical bone more, similar to the lumbar cortical bone trajectory screw to have a better inserted torque [30]. In addition, similar screw length in the present study may be one of the reasons for this phenomenon. In a word, in our FEA study, C1PS-C2IS was the most ideal construct with reference to the ROM, the stress on the implants and pullout strength.

### Limitations

C2 pedicle screw is widely used in the clinical application as a standard fixation technique for the posterior approach. Since there have been a plenty of biomechanical studies comparing pedicle screw with laminar screw, spinous screw and transarticular screw [31, 34], the present study used pedicle screw as a standard fixation method and only compared the biomechanical characteristics with the isthmus screw and short isthmus screw. Simultaneously, this study has the following shortcomings: (i) The data of the study from one healthy adult volunteer, and it wasn't possible to verify whether it differs from other comparisons, and more samples need to be included in the subsequent study to draw more thorough conclusions; (ii) Muscles are considered to be an important stabilizing cause of the vertebral joints and are thought to be important biomechanical stabilizing structure of the human body, but the three-dimensional finite element analysis model only reconstructed the vertebral body, intervertebral discs and ligaments, and could not analyse the influence of the muscles on biomechanics. (iii) This study is only a simulated biomechanical study, and its specific biomechanical properties should be compared with cadaveric specimen experiments to validate and complement this aspect of the study in order to provide a theoretical basis for clinical application.

In conclusion, C2 isthmus screw fixation provides sufficient stability. Thus, C2 isthmus screw fixation may be a biomechanically favourable option in cases with AAD and can be used as an effective C2 internal fixation method. However, future clinical studies are needed to evaluate the clinical outcomes of this technique.

### Rights and permissions

This article is licensed under a Creative Commons Attribution 4.0 International License, which permits use, sharing, adaptation, distribution and reproduction in any medium or format, as long as you give appropriate credit to the original author(s) and the source, provide a link to the Creative Commons licence, and indicate if changes were made. The images or other third party material in this article are included in the article's Creative Commons licence, unless indicated otherwise in a credit line to the material. If material is not included in the article's Creative Commons licence and your intended use is not permitted by statutory regulation or exceeds the permitted use, you will need to obtain permission directly from the copyright holder.

### Supplementary Information

**Supplementary Material 1.**

## Data Availability

The datasets and materials supporting the conclusions of this article are included within the article, and further inquiries can be directed to the corresponding author.
