# Supplementary material for: The C2 isthmus screw provided sufficient biomechanical stability in the setting of atlantoaxial dislocation-a finite element study
Source: BMC Musculoskelet Disord. 2024 May 29;25:423. doi: 10.1186/s12891-024-07470-6 (PMC11138020; doi:10.1186/s12891-024-07470-6)
Supplement: Supplementary file 1 — Supplementary Material 1. [file 12891_2024_7470_MOESM1_ESM.docx]

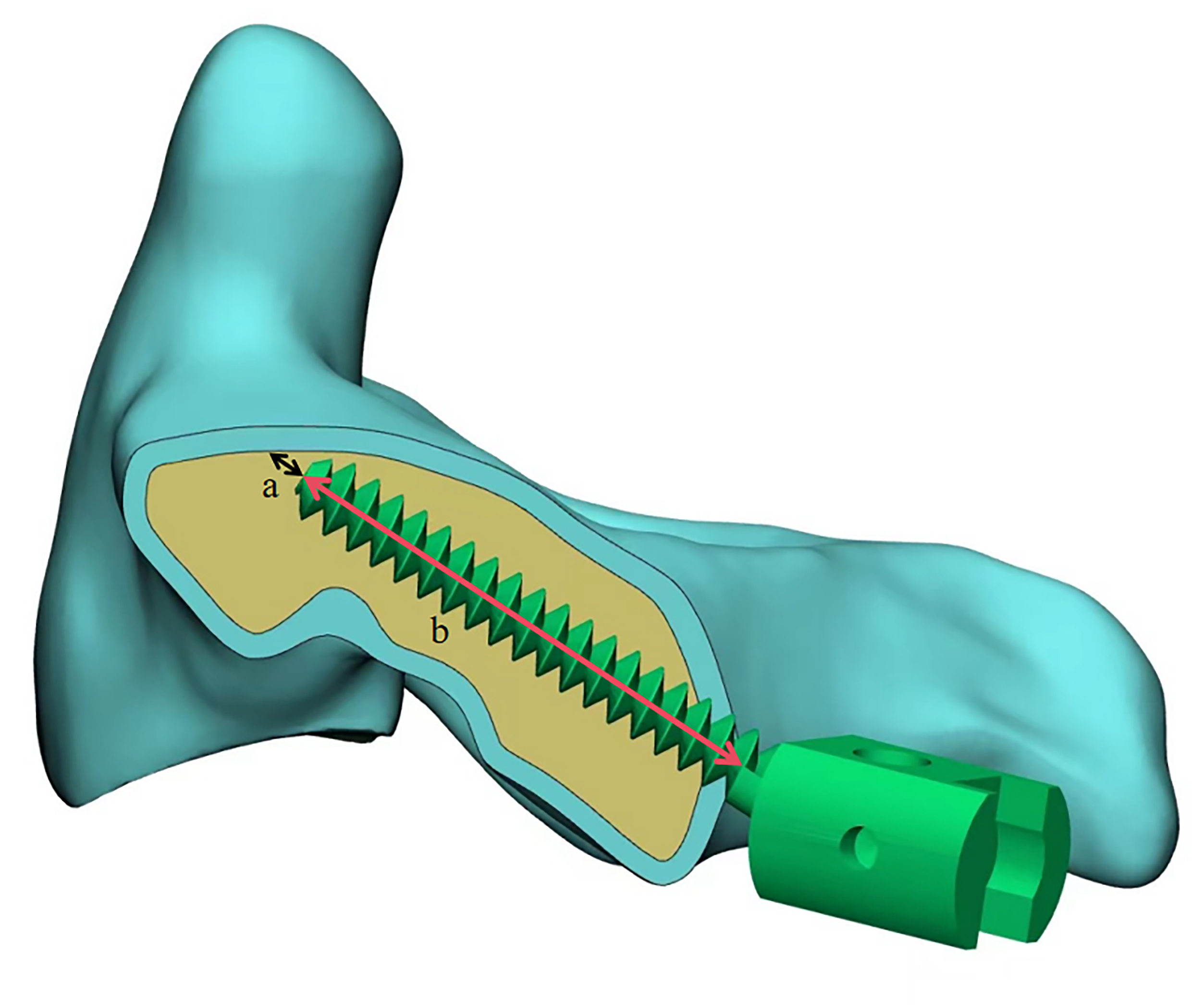


Supplymentary Fig 1 (C2 pedicle screw profile), the black arrow a represent the distance from the screw head to the bone cortex at the anterior margin, and the length is 1.7 mm; The black arrow b represent length of the pedicle screw, and the length is 26 mm;


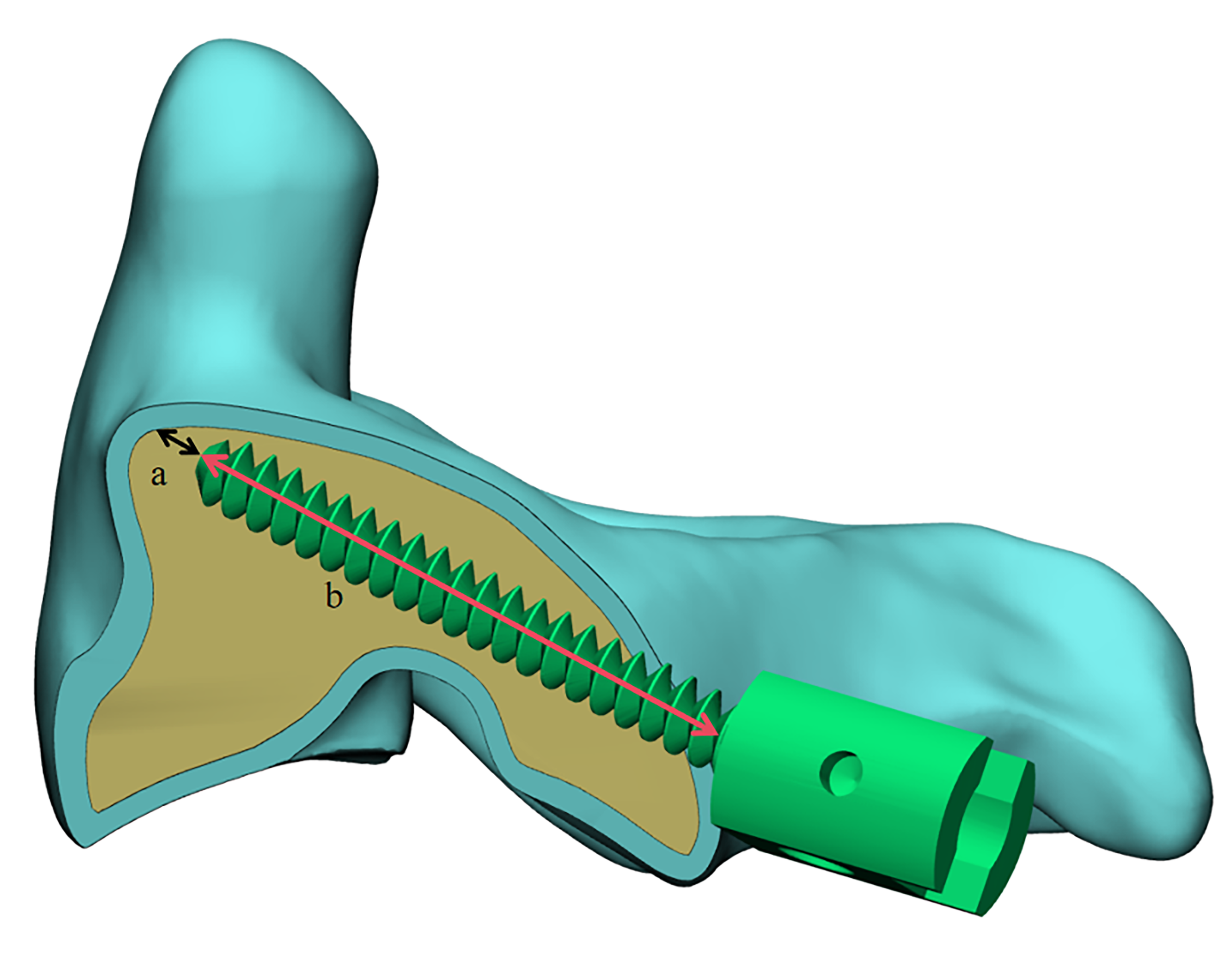


Supplymentary Fig 2 (C2 isthmus screw profile), the black arrow a represent the distance from the screw head to the bone cortex at the anterior margin, and the length is 1.2 mm; The black arrow b represent the length of the isthmus screw, and the length is 24 mm;


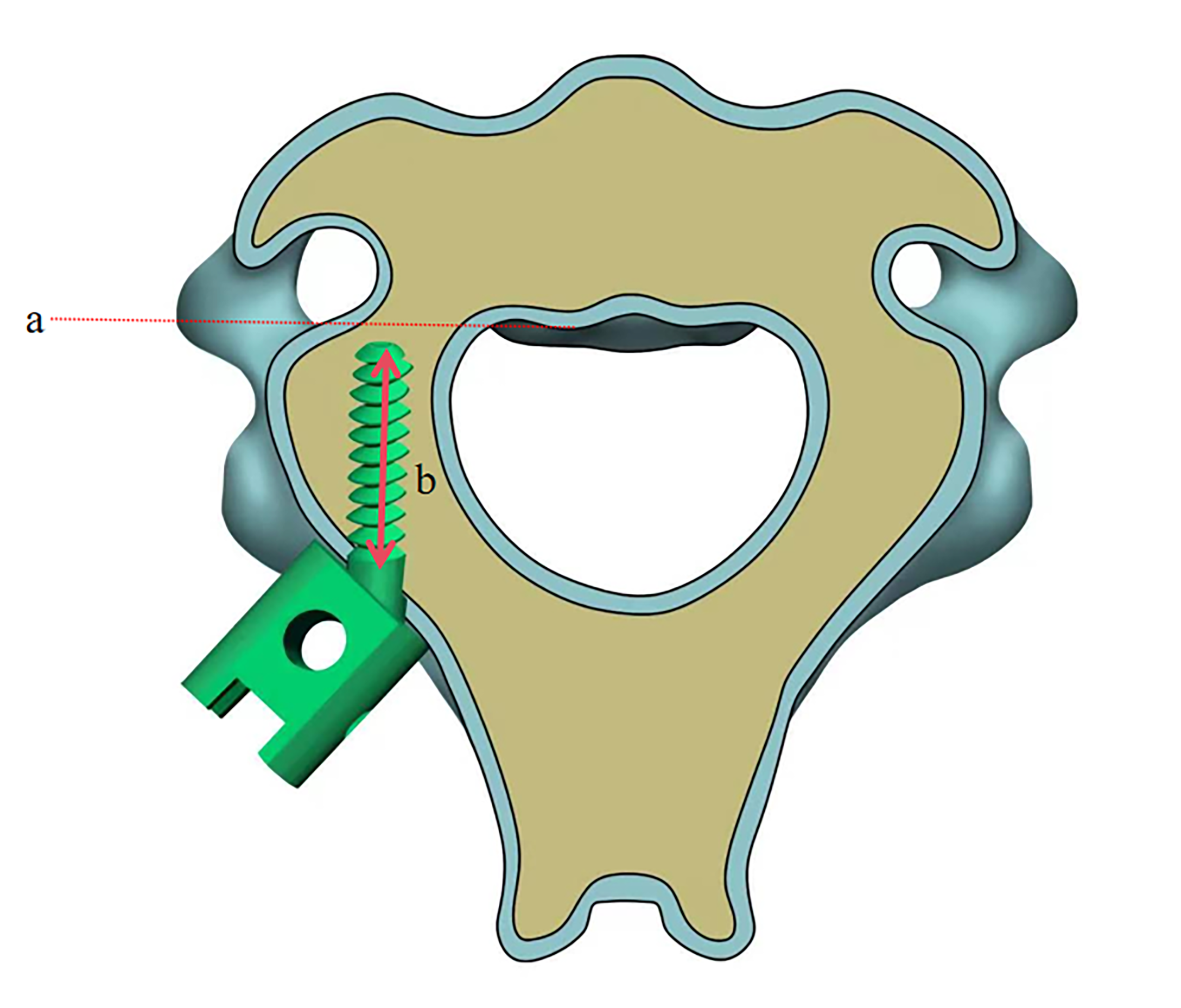


Supplymentary Fig 3 (C2 short isthmus screw profile), the red dotted line a represent the posterior wall of the transverse foramen; The red arrow b represent the length of short isthmus screw, and the length is 16mm;
